# Honey Compounds Exhibit Antibacterial Effects Against *Aggregatibacter actinomycetemcomitans* JP2

**DOI:** 10.3390/antibiotics14090887

**Published:** 2025-09-03

**Authors:** Abdelhadi Hbibi, Amine Ezzahi, Ferhat Ozturk, Niels A. J. Cremers, Jalal Kasouati, Ahmed Moussaif, Anders Johansson, Mimoun Zouhdi, Babacar Touré, Itto Maroui

**Affiliations:** 1 Department of Periodontology, International Faculty of Dental Medicine, College of Health Sciences, International University of Rabat, Sala Al Jadida 11100, Morocco; 2 Laboratory of Materials, Membranes, and Environment, Faculty of Science and Technology, University Hassan II of Casablanca, Casablanca 20000, Morocco; 3 Department of Biology, Health, and Environment, University of Texas, San Antonio, TX 78249, USA; 4 Department of Natural and Behavioral Sciences, Sul Ross State University, Eagle Pass, TX 78852, USA; 5 Department of Gynecology and Obstetrics, Maastricht University Medical Centre, 6202 AZ Maastricht, The Netherlands; 6 Triticum Exploitatie BV, 6222 NK Maastricht, The Netherlands; 7 Laboratory of Biostatistics and Epidemiological Research, Faculty of Medicine and Pharmacy, Mohammed V University in Rabat, Rabat 10000, Morocco; 8 Biotechnology and Engineering of Biomolecules Unit, National Centre for Nuclear Energy, Science and Technology (CNESTEN), Rabat 10001, Morocco; 9 Division of Molecular Periodontology, Department of Odontology, Umeå University, 901 87 Umeå, Sweden; 10 Department of Microbiology, Faculty of Medicine and Pharmacy, Mohammed V University in Rabat, Rabat 10000, Morocco; 11 Department of Restorative Dentistry and Endodontics, International Faculty of Dental Medicine, College of Health Sciences, International University of Rabat, Sala Al Jadida 11100, Morocco; 12 Research Laboratory in Oral Biology and Biotechnology, Department of Basic Sciences, Faculty of Dental Medicine, Mohammed V University in Rabat, Rabat 10000, Morocco

**Keywords:** *Aggregatibacter actinomycetemcomitans* JP2, periodontal diseases, honey, royal jelly, antibacterial agents, antimicrobial resistance

## Abstract

**Background:** *Aggregatibacter actinomycetemcomitans* JP2 genotype is a virulent pathogen linked to severe periodontitis and systemic diseases. Honey and royal jelly (RJ) have demonstrated bioactive properties against this microorganism. This study aims to assess the bioactive properties of honeys and RJ against this key periodontal pathogen and to preliminarily identify key compounds with antibacterial potential. **Methods:** The antibacterial activity of honeys and commercial products (manuka, L-Mesitran^®^ as a medical-grade honey-based formulation (MGHF), and Honix^®^ RJ) against *A. actinomycetemcomitans* JP2 was evaluated using the agar well diffusion method and microdilution assays. Extensive physicochemical characterization (e.g., hydrogen peroxide level, total phenolic content, and total flavonoid content) was conducted to correlate the bioactive compounds with the antimicrobial activity. **Results:** All tested samples exhibited varying antibacterial potency, with inhibition zones ranging from 21 to 37 mm. The MICs ranged from 40.7 to 104.3 mg/mL. MGHF, RJ, and multifloral honeys showed the lowest MICs. The pH of six out of eight samples could not induce enamel decalcification while the pH of three samples may not influence cementum demineralization. Vitamin C, zinc, magnesium, and potassium were present in measurable quantities, and were not associated with significant antibacterial activity. MGHF showed the highest hydrogen peroxide activity and TFC values. TFC and H_2_O_2_ content were statistically correlated with lower MIC values. **Conclusions:** Honey and RJ showed antibacterial activity against *A. actinomycetemcomitans* JP2, partly attributed to their content of hydrogen peroxide and flavonoids. Clinical trials are needed to confirm the potential role of honey, RJ, and their bioactive compounds in managing periodontitis.

## 1. Introduction

Periodontal diseases are biofilm diseases whose biofilm may harbor numerous pathogens that remain protected by this structure against host immunity and antimicrobial agents. These microbial agents trigger and sustain reactive inflammation in the periodontal tissues and contribute to chronic low-grade inflammation [1]. Among these pathogens, *Aggregatibacter actinomycetemcomitans*, considered a key periodontal pathogen, [2] is associated with advanced periodontal destruction [3]. This virulent microorganism belongs to the HACEK bacterial group (*Haemophilus* spp., *Aggregatibacter actinomycetemcomitans*, *Cardiobacterium hominis*, *Eikenella corrodens*, *Kingella kingae*), and was reported to be implicated in the pathogenesis of infectious endocarditis [4,5]. Other systemic conditions have also been associated with *A. actinomycetemcomitans* infection such as rheumatoid arthritis [6,7], atherosclerosis [8], and mood and stress-related disorders [9]. *A. actinomycetemcomitans* is primarily localized in the oral cavity and exists in various serotypes, some of which may be present even in individuals with a healthy periodontium [10]. Serotype b, and particularly the JP2 genotype, has gained specific attention due to its significant association with the initiation and progression of aggressive forms of periodontitis [11]. The JP2 genotype of *A. actinomycetemcomitans* was reported to be responsible for 10–20 times higher secretion of leukotoxins than the non-JP2 genotypes, while current evidence supports the link between the leukotoxicity of *A. actinomycetemcomitans* and the severity of periodontal destruction [12,13]. *A. actinomycetemcomitans* leukotoxin (LtxA) may help the bacterium to survive by destroying gingival crevice leukocytes, resulting in the suppression of local immune defenses [14].

Periodontal therapy is essentially based on the principle of biofilm disruption, supplemented by local and/or systemic antimicrobials if necessary. The adjunctive effect of these agents remains crucial to control periodontal infection and to overcome the limitations of subgingival debridement. The invasive ability of some periopathogens across the epithelial barrier, including *A. actinomycetemcomitans*, justifies the adjunction of systemic antibiotics, especially among young patients with severe periodontitis [15,16]. However, the indiscriminate use of antimicrobials globally has increased the emergence of multidrug-resistant bacteria. Compounding this issue is the limited introduction of new molecules by the pharmaceutical industry [17].

Antimicrobial resistance in dentistry (AMR) is mainly driven by the unjustified use of antimicrobials in the management of oral infections. In the United States, dentists are responsible for 10% of prescribed antibiotics for human infections and are the top specialty prescribers [18]. Similarly, in the United Kingdom, 100% of *A. actinomycetemcomitans* isolates are resistant to amoxicillin and metronidazole, 87.5% to clindamycin, and 83.9% to amoxicillin [19]. Thus, the growing AMR justifies new research directions on antimicrobials based on innovative agents and/or natural products such as bee products.

Among the apicultural derivatives under investigation in clinical microbiology, honey has demonstrated efficacy against various multi-resistant bacterial species and single-species biofilms [20]. Moreover, many oral bacteria have shown sensitivity to this product [21], including cariogenic bacteria [22] and periodontal pathogens such as planktonic *Porphyromonas gingivalis* and *P. gingivalis* in vitro biofilm [23], *Fusobacterium nucleatum* [24], and *Campylobacter* spp. [25,26]. Furthermore, Manuka honey was shown to prevent biofilm growth, and exert anti-plaque activity, and may help control gingivitis in patients undergoing orthodontic treatment [27].

In terms of oral tissue healing, post-extraction intra-socket administration of medical-grade honey has been reported to prevent local infection, promote healing, and reduce inflammation in animal models [28]. While the antibacterial potential of honey has been well-documented against several oral pathogens, research specifically targeting *Aggregatibacter actinomycetemcomitans*—and particularly its highly virulent JP2 genotype—remains limited. Additionally, few studies have employed well-characterized honey samples with standardized methodologies, which complicates the interpretation and comparison of results across studies. This contributes to an important gap in the current understanding of honey’s therapeutic potential against periodontal pathogens and highlights the need for more focused and standardized investigations in this area.

To address this gap, this study aims to investigate the in vitro susceptibility of *A. actinomycetemcomitans* JP2 to selected honeys and royal jelly. Additionally, it seeks to characterize the physicochemical properties of these natural products to preliminarily identify specific compounds that may contribute to their antibacterial activity against this key periodontal pathogen.

## 2. Results

### 2.1. Antibacterial Activity

The agar well diffusion method revealed the antibacterial activity of all tested products against the CCUG 56172 strain. The largest inhibition zone (IZ) for *A. actinomycetemcomitans* was observed following the application of H4 honey, chlorhexidine, and subsequently H6, H5, H1, H7, H3, and H2 honeys. The smallest IZ was recorded around H0, while no inhibition diameter was observed around distilled water (Table 1).

The microdilution test affirmed antibacterial activity for all products with different MIC values ranging from 40.7 to 104.3 mg/mL. The lowest MICs were noted with H0, H2, H5, and H1, respectively. MBCs were equal to or slightly above the MICs. The MBC/MIC ratio was strictly less than 4 (between 1 and 1.07) and underscores the bactericidal nature of the tested samples.

### 2.2. Physicochemical Analysis

The pH values of the examined products indicate acidity, ranging from 3.74 to 4.20. The most acidic honey in the studied selection is H7 (Eucalyptus honey), while the top three least acidic honeys were represented, respectively, by H1, H0, and H4 (bupleurum, cedar, and euphorbia). The pH values of the dilutions corresponding to the MICs were between 5.12 and 7.63 (multifloral honeys H4 and H5, respectively). The free acidity values of the analyzed honeys varied from 10 to 23 mLEq/Kg. The most increased free acidity was recorded in H0 (Table 2).

Moisture is a maturity-related parameter crucial for honey stability during storage, with obtained values ranging from 18.83% to 21.59%. The ash content in the analyzed samples varied between 0.14% and 0.33%, with the highest ash concentration found in mountainous multifloral honey M4 (bupleurum, cedar, and euphorbia) (Table 2).

The mineral content showed heterogenous results among samples. Apart from H0 and H2, zinc content was under the limit of detection. The highest magnesium and potassium level was registered with H4, while the lowest content for these minerals was noted with H6 and H3, respectively (Table 3).

### 2.3. Antioxidant, Hydrogen Peroxide Activity, and Vitamin C Content

#### 2.3.1. Antioxidant Compounds

Total phenolic content (TPC) ranged between 1.20 mg/kg honey and 434.30 mg/kg honey, while Total Flavonoid Content (TFC) ranged between 4.42 mg/kg and 114.01 mg/kg. Moreover, H0 showed the highest hydrogen peroxide activity, phenolic and flavonoid content, and the darkest color (dark amber). Vitamin C content ranged between 1.59 and 13.44 among samples. H2 registered the highest content followed by H1, H3, and H5 (Table 4).

#### 2.3.2. Antioxidant Activity and Mineral Content

Radical scavenging activity was highly registered with H4 and H1, respectively, while H0 showed the lowest DPPH activity followed by H3.

The mineral content showed heterogenous results among samples. Apart from H0 and H2, zinc content was under the limit of detection. The highest magnesium and potassium level was registered with H4, while the lowest content for these minerals was noted with H6 and H3, respectively (Table 3).

Pearson correlation coefficient showed a significant negative correlation between MIC values and hydrogen peroxide activity and total flavonoid content (Table 5).

## 3. Discussion

*A. actinomycetemcomitans* is one of the major periodontal pathogens whose synergistic effect with other germs leads to significant destruction of periodontal tissues [29]. Furthermore, the JP2 strain of this species showed particularly significant virulence, initiating progressive tissue damage and leading to severe periodontitis and tooth loss [30,31]. Its specific virulence is attributed to various factors, including exotoxins (leukotoxin Ltxa and cytolethal distending toxin), endotoxins (lipopolysaccharides), and cytokine binding molecules [32]. Considering the increasing resistance of this periodontal and HACECK germ to antibiotics, in a global AMR context, the need to enlarge its bacterial sensitivity is highly requested.

In terms of antibacterial activity, all tested honey/RJ samples demonstrated antibacterial efficacy against *A. actinomycetemcomitans* JP2 (CCUG 56172). Similarly to previous studies which preliminarily assessed the antibacterial potency of honey using agar well diffusion [33,34,35,36], the current work explored the sensitivity of the JP2 strain to honeys/RJ, using the same method as primary antibacterial assessment. The agar well diffusion method was employed instead of discs, to enhance honey diffusion within the agar [37]. Consequently, mountainous multifloral honey (H4, sourced from euphorbia, bupleurum, and cedar) exhibited the largest IZ, followed by thyme, manuka, eucalyptus, multifloral (H5), rosemary honey, and RJ. The IZ of multifloral honey (H4) surpassed that of chlorhexidine at 0.12%, while both (H4) and thyme (H6) displayed larger diameters than those of manuka and MGHF, with the latter presenting the smallest IZ. However, the small IZ for H0 might be related to the fat component (lanoline) in the formulation which could interfere with the dissolving in agar, limiting the assay significance for this product. The ratio of MBC/MIC was around 1 for all samples, which indicates the bactericidal activity of studied products. The concordance between MIC and MBC values may be attributed to the use of visual determination of color change following TTC (triphenyl tetrazolium chloride) addition to inoculated honey dilutions, which was confirmed further by cultures on Brain Heart Infusion (BHI) agar. The inoculation was carried out from the three wells corresponding to the lowest dilutions of honey which showed no color change after TTC addition of BHI agar plates (Oxoid^TM^) to confirm the total absence of bacterial viability.

The difference in botanical origin of the honey samples can partly explain the variety of recorded IZs. Indeed, euphorbia honey has shown its effectiveness, alone and in combination with other plants, against microorganisms commonly found in infected wounds [38]. This can be attributed to its high content of phenolic compounds and its strong inhibitory and antioxidant enzymatic activity [39,40]. Concerning manuka honey, its antimicrobial properties are well-documented, and its antibacterial potency is mainly linked to the high methylglyoxal content [41,42]. Depending on the bacterial species, this honey can modify or alter the shape of the target bacterium and alter its septal ring which is involved in cell division [43]. As for eucalyptus honey, several studies have demonstrated its antibacterial activity against *Staphylococcus epidermidis*, *Staphylococcus pyogenes*, *Staphylococcus aureus*, and *Klebsiella pneumoniae* [44]. The present work outlined the bacterial sensitivity of an additional virulent germ (*A. actinomycetemcomitans* JP2). The mechanisms of such antimicrobial activity are still unclear but seem to be linked to the presence of eucalyptus essential oil. Thyme honey has also shown through this work an obvious antibacterial activity against *A. actinomycetemcomitans*, marking additional evidence of its activity against this pathogen. Despite its documented clinical benefits, particularly in relieving cough and treating mucositis and post-radiation xerostomia, thyme honey remains under-investigated for antimicrobial purposes in the oral cavity [45,46].

Concerning MICs and MBCs, the medical-grade honey-based formulation (H0) exhibited the lowest MIC, followed by multifloral honey (H5), Manuka honey, then multifloral honey (H4). Furthermore, the assessed RJ demonstrated efficacy comparable to manuka honey. The royal jelly studied by Kholsa et al. against *A. actinomycetemcomitans serotype a* (ATCC 29523) lacked a comparison to any control product [47]. The discrepancy in the antibacterial potency of honey samples between the agar diffusion test and microplates could be explained by a possible difference in dissolution and diffusion of the honeys in agar due to their high content in macromolecules. This discordance shows the interest in interpreting with caution the inhibition zones’ diameters of honeys on agar and the need to confirm experiment results by the microdilution test [48].

During processed experiments, honey samples were not heated to avoid macromolecule and vitamin denaturation. Specific syringes were used to pipette these products and compensate for their high viscosity. Furthermore, this precaution was dictated by the expected reduction in antibacterial honey activity previously documented [49,50]. In this context, some key antimicrobial compounds in honey, such as vitamin C, are known to be sensitive at high temperature [49].

The lowest MIC observed with the medical-grade honey-based formulation (H0) may be attributed a priori to its supplementation with vitamin C and other bioactive components [51]. Indeed, this micronutrient has previously been associated with specific antibacterial properties [51]. However, vitamin C analysis of the studied samples revealed a high concentration in H2, whereas H0 showed a level comparable to that of H6. The lack of correlation between antibacterial potency and vitamin C content among the samples may be due to the instability of vitamin C at the experimental incubation temperature (37 °C), which could limit its antibacterial effect against the tested strain. In contrast, other studies have shown that vitamin C, when combined with honey, can enhance its antibacterial activity against both planktonic and biofilm-forming bacteria [49]. Furthermore, magnesium, potassium, and zinc content among studied products did not affect significantly their antibacterial activity toward the assessed strain. These minerals, more specifically magnesium and zinc, were documented for their role in antimicrobial activity [52].

Hydrogen peroxide activity was notably high in the top three antibacterial samples in the current study (H0, H2, and H5, respectively). Moreover, hydrogen peroxide levels were correlated with the lowest MIC values, indicating the highest antibacterial potency. This correlation may help explain the differences in antimicrobial efficacy observed among the studied products. This compound is considered the main antibacterial ingredient in honey, and the reason for its concentration variability among honeys is still under-researched [53]. However, numerous studies confirmed the validity of the positive association between the concentration of hydrogen peroxide in honey and its antimicrobial activity [54,55,56]. Phenols and, more specifically, flavonoids contents, are highly present in the top two antimicrobial samples (H0 and H2) and are significantly correlated with antibacterial activity (MICs values) in these products. Polyphenols were reported to exhibit pro-oxidant activity, which is considered as an alternative pathway of H_2_O_2_ formation in honey [53].

In the applied methods of this study, the microdilution test on a 96-well microplate was performed using BHI Broth instead of Mueller–Hinton (MH) broth. This substitution was justified by a weak or absent growth of the studied bacterium in certain repetitions when using Muller Hinton broth. The CLSI (Clinical and Laboratory Standard Institute) M45 guideline suggests the use of HTM broth (Haemophilus Test Medium) or CAMHB-LHB (cation-adjusted Mueller–Hinton broth supplemented with lysed horse blood) [57] instead of MH for studying antimicrobials MICs for HACEK group, including *A. actinomycetemcomitans*. However, due to the unavailability of these recommended broths, the choice to employ BHI broth for MIC tests was primarily based on its notable support for the growth of this strain. Previous studies have successfully used BHI broth as a nutrient-rich medium for determining the MICs of antibacterial agents against *A. actinomycetemcomitans*. In fact, Zhang et al. utilized BHI broth to assess the antibacterial effect of erythritol against both *A. actinomycetemcomitans* and *P. gingivalis* [58]. Similarly, BHI infusion was employed to evaluate the sensitivity of the *A. actinomycetemcomitans* reference strain ATCC 43718 to lemongrass oil [59]. Other studies exploring the efficacy of essential oils against this microorganism have also utilized BHI due to its micro-nutritional benefits [60].

The physicochemical analysis showed values of moisture, electrical conductivity, ash rate, total acidity, and pH in accordance with the standards of the Codex Alimentarius [61]. Such results testify to the biochemical stability of the tested products and the absence of undesirable fermentation. Furthermore, the pH values of the dilutions corresponding to the assessed MICs were beyond the critical threshold for carious decalcification of the enamel (pH of 5.5) [62], except for H2 (royal jelly) and H5 (multifloral honey) (pH 5.14 and 5.12, respectively). However, the samples whose MIC pH is higher than the critical threshold for demineralization of cementum-pH of 6.7 [63] are H3 (Rosemary honey), H4 (multifloral honey based on bupleurum, cedar, and euphorbia) and H7 (Eucalyptus honey). Thus, these last three products would remain harmless—in terms of decalcification—with respect to enamel and cementum during a possible clinical application. The other samples would require the addition of selected additives to increase their pH values before any clinical use.

## 4. Materials and Methods

### 4.1. Bacterial Strain

The reference strain CCUG 56172 delivered by the “Culture Collection University of Gothenburg” (CCUG)–Sweden was used to assess the antibacterial susceptibility of *A. actinomycetemcomitans* to honey and royal jelly. This strain, also known under the reference HK921 in other international microbial collections, represents *A. actinomycetemcomitans* serotype b, JP2 genotype. It was delivered in freeze-dried form and prepared for culture following the CCUG instructions. The subculture was carried out preferentially on a selective culture medium for *A. actinomycetemcomitans*, Dentaid-1 [64]. The cultured germ was incubated in jars at 5% CO_2_ at 37 °C for 48 h.

### 4.2. Tested Products

Selected local honeys from a single beekeeper (Jasmine Cooperative, Fes, Morocco), as well as honeys of foreign origin and a royal jelly (Honix^®^) from the Pharmalink S.L pharmaceutical laboratory (Barcelona, Spain) were included in this study (Table 6).

### 4.3. Antibacterial Activity

Preliminary antibacterial screening was conducted using the agar well diffusion method. Products that exhibited inhibition zones were subsequently evaluated by determining their Minimum Inhibitory Concentrations (MICs) and minimum bactericidal concentrations (MBCs), in line with established protocols for assessing the antimicrobial activity of honey and other natural products [48,65,66].

For agar well diffusion assay on *A. actinomycetemcomitans*, a bacterial suspension was prepared from a fresh culture (24 h) with a turbidity of 0.5 McFarland adjusted by a nephelometer (Sensititre^®^ Nephelometer, Waltham, MA, USA). This suspension was made in a 0.9% saline solution. Agar surface was inoculated using the bacterial suspension previously prepared by swabbing. Then, wells were created of 5 mm in diameter which were filled with the undiluted honeys and royal jelly as well as the negative and positive controls, to the tune of 100 μL each. These controls represented, respectively, sterile distilled water and 0.12% Chlorhexidine (Perio-Aid^®^ Intensive Care, Barcelona, Spain). The prepared Petri dishes were incubated at 37 °C under 5% CO_2_ for 48 h. The inhibition zone (IZ) of each honey/RJ sample was measured using a digital caliper as the whole diameter of each clear zone (including the well) was recorded in millimeters. All experiments were performed aseptically and in triplicate.

MICs and MBCs were determined using a microdilution assay with 96-well microtiter plates as previously described for natural products including bee products [48,65,66]. For this purpose, we prepared a bacterial suspension with a turbidity of 0.5 McFarland (approximately 1.5 × 10^8^ CFU/mL) from a fresh culture. Then, 1 mL of this suspension was added to 9 mL of BHI Broth (Brain Heart Infusion, Oxoid^TM^, Waltham, MA, USA) to obtain an approximate final concentration of 1.5 × 10^7^ CFU/mL. The tested honeys/RJ were diluted aseptically in sterile distilled water at concentrations ranging from 1 to 15%, which was predicted based on previous studies [47,48,65,66]. To each well, 80 μL of honey dilution was added, then 80 μL of the previously prepared bacterial suspension was added. The positive control well contained 80 μL of the amoxicillin (AMX) solution at a concentration of 10 mg/mL, and 80 μL of the bacterial suspension. The negative control well contained 160 μL of the BHI broth. The prepared microplates were incubated for 18 to 24 h, at 37 °C under 5% CO_2_ in static conditions.

After 24 h, 40 µL of 1% triphenyl tetrazolium chloride (TTC) solution (Oxoid^TM^) was added to each well as a cell viability indicator solution, and the microplate was re-incubated for 2 h at 37 °C under 5% CO_2_. Positive bacterial growth in the wells was determined by a visual color shift in their contents towards red or the presence of faintly red deposits at the well’s bottom. MIC, defined as the lowest concentration of honey/RJ at which no bacterial growth is observed, was indicated by the absence of color change.

To determine the MBC, the inoculation was carried out—from the 3 wells corresponding to the lowest concentrations of honey/RJ which showed no color change after TTC addition—on BHI agar plates (Oxoid^TM^) to confirm the total absence or very low (<0.01%) bacterial viability. The MBC was identified as the lowest concentration of honey that effectively killed at least 99.99% of the bacterial population.

To elucidate the antibacterial activity of the tested products, the MBC/MIC ratio was computed for each. A ratio below 4 denoted bactericidal activity, while a ratio exceeding 4 indicated a bacteriostatic effect, following established guidelines [67].

### 4.4. Physicochemical Analysis

The assessment of pH, free acidity, humidity level, and ash content followed the methodology outlined by Bogdanov et al. [68]. pH measurement involved using a calibrated pH meter according to the Bogdanov protocol. Electrical conductivity was determined using a conductivity meter, based on the measurement of electrical resistance at 20 °C. The pH values were measured for both undiluted honeys and those diluted to concentrations corresponding to the MICs. The free acidity was calculated by assessing the needed quantity of hydroxides ions (in millimoles) to be added to bring 1 kg of honey to a pH equal to 7. The humidity was determined by measuring the refractive index at 20 °C using an Atago type refractometer. As for the ash rate, it was assessed using the gravimetric method by incinerating 5 g of honey in an electric oven at 600 °C for 3 h.

The Beretta et al. (2005) method was followed to determine the color of honey samples [69]. A 10% honey solution was prepared using 100 mg of each honey sample and 1000 μL warm DI water (50 °C). Honey samples were vortexed and centrifuged for 5 min at 14,000 RPM. Of each honey sample, 200 μL was placed into a 96-well plate as triplicates and DI water was used as the blank. The absorbances of the honey samples were read at 560 nm using the SpectraMax ABS Plus Spectrophotometer (Molecular Devices, San Jose, CA, USA). The absorbance values of each sample were used to determine the color of the honey samples according to the USDA color standard designations (Table 7) as Abs560 was multiplied by a factor of 3.15.

The mineral characterization of tested samples used atomic absorption spectrometry (AAS) to identify magnesium potassium and zinc load. Then 1 g of each sample was placed in a digestion flask and dissolved by 10 mL nitric acid (HNO_3_), then heated to evaporate acid excess and concentrate the solution. The obtained concentrate was filtered using cellulose filter and diluted to a volume of 50 mL with distilled water to obtain a concentration of analytical elements suitable for analysis. The analysis followed the spectrometer parameters and used the calibration method based on the prepared standards.

### 4.5. Antioxidant Properties

#### 4.5.1. Total Phenolic Content (TPC)

The TPC assay was based on the reaction with the Folin–Ciocalteu’s (FC) reagent using 10% (*w*/*v*) honey/RJ dilutions (distilled water) and diluted gallic acid in methanol/water (1:1). Then 100 μL of each gallic acid standard solution, honey/RJ samples, and distilled water were transferred to the microfuge tubes and incubated for 2 h in the dark at room temperature (RT) (25 °C). Then 200 μL of solution from each tube was transferred to the 96-well plate in triplicates and the absorbance was read at 765 nm.

#### 4.5.2. Total Flavonoid Content (TFC)

The TFC Assay method was based on aluminum complex production in an alkaline medium, detecting the absorbance at 510 nm. The catechin was used as the reference component and the standard solutions were prepared as 20, 40, 60, 80, and 100 μg/mL as 500 μL final solution. Then 200 μL of each preparation was transferred to a 96-well plate in triplicates and absorbance was measured at 510 nm. The results were expressed as mg catechin equivalents (CEQ) per kg of honey/RJ.

#### 4.5.3. DPPH Radical Scavenging Assay (RSA)

The present study used the 2,2-difenil-1-picril-hidrazil (DPPH) radical-scavenging assay to measure the antioxidant activity in the honey/RJ samples according to Brand-Williams method [70]. The prepared plates were read at 517 nm and absorbance values were used to determine the reduction in the DPPH radical. The radical-scavenging activity (RSA) was determined as a percentage of DPPH discolor using the following formula: % RSA = ([AB − AA]/AB) × 100, where AB is the absorbance of the mixture when the sample extract is added at a certain quantity and AA is the absorbance of the DPPH solution.

#### 4.5.4. Hydrogen Peroxide Activity

The activity of hydrogen peroxide in honey samples was measured using a colorimetric assay that utilizes horseradish peroxidase (HRP) to catalyze the oxidation of o-dianisidine by H_2_O_2_, forming a stable purple product detectable at 550 nm. Honey/RJ samples were diluted to 33% (*w*/*v*) and incubated at 35 °C for 4 h in a shaking incubator at 180 RPM. Each sample, H_2_O_2_ standard, and PBS blank were transferred in triplicates (40 µL each) into a 96-well microplate. A 135 µL aliquot of colorimetric reagent was added to each well, and the mixture was incubated at RT for 5 min. The reaction was stopped by adding 120 µL of 6 M H_2_SO_4_ to all wells. H_2_O_2_ activity in honey/RJ samples was quantified using the linear portion of the H_2_O_2_ standard curve and expressed in micromolar (µM) units.

#### 4.5.5. Vitamin C Analysis

Isocratic reversed-phase high-performance liquid chromatography (RP-HPLC) was used to identify and quantify vitamin C in tested samples (UHPLC UltiMate 3000; Thermo Fisher Scientific, Waltham, MA, USA). This technique used UV detection at 254 nm and was based on elution with potassium dihydrogen phosphate buffer at pH 2.92; methanol (95:5, *v*/*v*) was used at a flow rate of 0.5 mL/min. The vitamin C analysis was performed by isocratic RP-HPLC method with UV detection at 254 nm. In this context, isocratic elution used potassium dihydrogen phosphate buffer at pH 2.92: methanol (95:5, *v*/*v*) at a flow rate of 0.5 mL min 1. Vitamin C identification was based on comparison of its UV spectrum and retention time with that of standard. Ascorbic acid content was calculated based on its calibration curve (0.05–1.00 mg/mL) and expressed as mg per kg honey.

#### 4.5.6. Statistical Analysis

IZ diameters, MICs, MBCs, physicochemical and antioxidant parameters, as continuous variables with a normal distribution, were expressed as mean ± standard deviation. One way ANOVA test was used to assess measured value differences among honey/RJ samples, followed by Tukey’s post hoc test for pairwise comparisons among IZ, MICs, and MBCs. Additionally, Pearson correlation coefficient was computed to examine relationships between MICs and mineral, phenolic, flavonoid, and hydrogen peroxide content. Statistical analysis was performed using SPSS software (version 18.0 for Windows and Mac OS X), with a significance level set at *p* < 0.05.

## 5. Conclusions

The present work demonstrates the antibacterial potential of various honey samples and royal jelly against the *A. actinomycetemcomitans* JP2 genotype (CCUG 56172 strain) in both agar well diffusion and microdilution assays. Hydrogen peroxide concentration appears to be the principal factor driving the variability in antibacterial activity toward the assessed JP2 strain. These findings align with previous reports indicating that honey’s antimicrobial activity is multifactorial, involving a synergistic interaction between hydrogen peroxide, phenolic compounds, and other yet-undetermined bioactive molecules. Further phytochemical investigations are warranted to isolate and characterize these compounds and elucidate their mechanisms of action, particularly in the context of biofilm resistance.

Any future therapeutic use of honey-based products in the oral cavity should, however, consider the complex biofilm organization of oral bacteria, which significantly impacts antimicrobial susceptibility, as well as the critical thresholds for dental hard tissue demineralization, to avoid iatrogenic lesions. Additionally, such formulations should be carefully designed to preserve the ecological balance of the oral microbiome, which plays a vital role in maintaining physiological homeostasis.

Finally, it would be beneficial to investigate the role of natural antimicrobial compounds in preventing the emergence of resistant strains, especially in patients suffering from systemic conditions such as rheumatoid arthritis or cardiovascular diseases, where *A. actinomycetemcomitans* plays a critical role. Future research in this area could not only enhance the management of periodontal infections but also help curb the overprescribing of local antimicrobials and promote more sustainable clinical practices.

## Figures and Tables

**Table 1:** Average of inhibition zones, MICs, and MBCs of tested products against the CCUG 56172 strain.

Samples	IZ (mm)	MIC (mg/mL)	MBC (mg/mL)
H0	21 ± 2.26 ^a, b^	40.70 ± 6.4 ^a, b, c, d^	40.70 ± 6.4 ^a, b, c, d, e, f^
H1	28.3 ± 3.78	71.25 ± 3.37 ^a, g, h, i^	71.25 ± 3.37 ^a, b, c, d, e, f^
H2	22.3 ± 2.08 ^c^	54.6 ± 8.93 ^j, k, l, m^	54.6 ± 8.93 ^c^
H3	25 ± 7.81	102.5 ± 4.33 ^b, g, j, n, o^	109.6 ± 4 ^a, b^
H4	37 ± 2.64 ^a, c^	100.75 ± 13.42 ^c, h, k, m, p^	100.75 ± 13.42 ^a, c^
H5	28.3 ± 8.02	71.05 ± 4.24 ^d, n, p, q^	70.56 ± 4.24 ^a, d, e, f^
H6	30.6 ± 2.08	79.05 ± 11.68 ^e, l, o, r^	78.79 ± 11.68 ^a, c, d, e^
H7	27.3 ± 3.05	104.3 ± 7.45 ^f, i, q, r^	106.3 ±7.4 ^a, b^
CHX	34.6 ± 2.51 ^b^	ND	ND

Values with the same letters (alphabets a to r) in the same column are significantly different according to Tukey’s post hoc test (*p* < 0.05). CCUG: Culture Collection University of Gothenburg. IZ: Inhibition zone; MIC: Minimum Inhibitory Concentration, MBC: Minimum Bactericidal Concentration; CHX: Chlorhexidine; ND: Not determined.

**Table 2:** Different values of the physicochemical analyzed items of honey samples and royal jelly.

Samples	pH	MIC pH ^(^*^)^	Free Acidity (mLEq/Kg)	Moisture (%)	Ash (%)	Electrical Conductivity (µs/cm)
H0	4.06 ± 0.06	6.29 ± 0.07	23 ± 0.06	18.97 ± 0.02	0.321 ± 0.001	237.00 ± 6.55
H1	4.20 ± 0.005	5.51 ± 0.03	15 ± 0.04	20.59 ± 0.01	0.333 ± 0.001	368.67 ± 0.57
H2	3.92 ± 0.02	5.14 ± 0.03	14.8 ± 0.02	19.23 ± 0.02	0.310 ± 0.001	500.00 ± 1
H3	3.76 ± 0.01	7.21 ± 0.08	12 ± 0.01	21.06 ± 0.04	0.298 ± 0.002	132.87 ± 0.96
H4	4.16 ± 0.01	7.63 ± 0.07	15 ± 0.02	19.45 ± 0.04	0.329 ± 0.002	306.67 ± 1.15
H5	3.78 ± 0.01	5.12 ± 0.02	20 ± 0.05	20.41 ± 0.01	0.188 ± 0.001	263.33 ± 1.15
H6	3.86 ± 0.01	5.60 ± 0.03	21 ± 0.05	18.83 ± 0.04	0.192 ± 0.002	334.33 ± 32.65
H7	3.74 ± 0.01	7.38 ± 0.05	15 ± 0.02	19.88 ± 0.01	0.143 ± 0.001	134.17 ± 1.04

(*) MIC pH refers to the pH of sample dilution at the Minimum Inhibitory Concentration.

**Table 3:** Magnesium, potassium, and zinc content in honey and royal jelly samples.

	Mg (mg/kg)	RSD (%)	K (mg/kg)	RSD (%)	Zn (mg/kg)	RSD (%)
H0	5.445	1.4	131.3115	0.6	0.105	10.2
H1	4.056	0.5	211.935	1.6	<DL *	_
H2	7.533	0.9	102.585	0.3	0.0285	18
H3	2.8995	1.1	42.462	0.1	<DL *	_
H4	6.2745	0.5	179.505	0.7	<DL *	_
H5	3.804	1.8	109.1325	0.3	<DL *	_
H6	2.5785	2.1	143.7375	0.4	<DL *	_
H7	5.4465	1.5	114.4605	0.1	<DL *	_

RSD: relative standard deviation; DL: detection limit, DL * = 0.0005 (mg/L).

**Table 4:** Hydrogen peroxide, antioxidant activity, vitamin C content, and color of tested products.

	H_2_O_2_ (µM)	TPC (GAE) mg/kg	TFC (CEQ) mg/kg	DPPH (RSA%)	Vitamin C mg/kg	Color Absorbance (Abs560)	USDA Standard Designation
H0	546.56 ± 0.159	434.30 ± 0.152	114.01 ± 0.031	22.0 ± 0.041	2.67 ± 0.17	4.54	Dark Amber
H1	62.33 ± 0.003	51.49 ± 0.002	4.42 ± 0.001	70.0 ± 0.003	7.01 ± 0.61	0.165	Extra Light Amber
H2	407.93 ± 0.007	36.28 ± 0.005	42.33 ± 0.013	54.0 ± 0.079	13.44 ± 0.87	0.081	White
H3	6.73 ± 0.001	1.20 ± 0.0005	5.49 ± 0.001	24.0 ± 0.0005	6.38 ± 0.72	0.094	White
H4	84.96 ± 0.00	122.95 ± 0.326	20.07 ± 0.003	78.0 ± 0.004	1.59 ± 0.11	0.352	Light Amber
H5	94.30 ± 0.002	73.10 ± 0.003	11.72 ± 0.002	39.0 ± 0.0008	4.95 ± 0.31	0.164	Extra Light Amber
H6	75.11 ± 0.002	104.91 ± 0.004	14.61 ± 0.006	57.0 ± 0.011	2.85 ± 0.13	0.257	Light Amber
H7	20.26 ± 0.001	37.25 ± 0.001	8.45 ± 0.001	39.0 ± 0.014	4.15 ± 0.21	0.123	Extra Light Amber

H_2_O_2_: hydrogen peroxide; TPC: Total Phenolic Content; GAE: gallic acid equivalents; TFC: Total Flavonoid Content; CEQ: catechin equivalents; DPPH: 2,2-Diphenyl-1-picrylhydrazyl; RSA: radical-scavenging activity.

**Table 5:** Pearson correlation between MIC and measured bioactive compounds in honeys/RJ samples.

	Mg	K	H_2_O_2_	TPC	TFC	DPPH	Vitamin C
MIC	−0.26 (0.527) ^c^	−0.1459 (0.73) ^c^	−0.86 (0.006) ^a^	−0.62 (0.10)	−0.75 (0.033) ^b^	0.1825 (0.6653) ^c^	−0.3126 (0.451) ^c^

^a^: Significant negative correlation between MIC and H_2_O_2_ content (r = −0.86, *p* < 0.01). ^b^: Significant negative correlation between MIC and TFC (r = −0.75, *p* < 0.05). ^c^: Absence of significant correlations were observed between MIC and magnesium, potassium TPC, DPPH, and vitamin C.

**Table 6:** Tested samples, their botanical and geographical origins as well as their harvest seasons.

Coding	Product	Botanical Origin	Geographic Origin	Harvest Season
H0	L-Mesitran^®^ (Medical-Grade Honey-based Formulation)	UD	The Netherlands/UD *	UD
H1	Mānuka Health	Manuka	New Zealand	UD
H2	Honix^®^ (Royal jelly)	UD	UD	UD
H3	Monofloral Honey (JC)	Rosemary	Midelt/Morocco	02/2021
H4	Multifloral Honey (JC)	Bupleurum, cedar and euphorbia	Timhdit + Tiznit/Morocco	09/2021
H5	Multifloral Honey (JC)	Multifloral	Had Kourt/Morocco	07/2021
H6	Monofloral Honey (JC)	Thyme	Timhdit/Morocco	08/2020
H7	Monofloral Honey (JC)	Eucalyptus	Guersiv/Morocco	05/2021

JC: Jasmine Cooperative; UD: Undefined; *: the product is manufactured in the Netherlands and honeys used in its formulation are collected from many other countries.

**Table 7:** Color designations of honey.

USDA Color Standard Designation	Color Range Pfund Scale (mm)	Sample Result Range
Water White	≤8	0–0.094
Extra White	>8 and ≤17	0.094–0.189
White	>17 and ≤34	0.189–0.378
Extra Light Amber	>34 and ≤50	0.378–0.595
Light Amber	>50 and ≤85	0.595–1.389
Amber	>85 and ≤114	1.389–3.008
Dark Amber	>114	>3.008

USDA Color Standard Designation Color Range Pfund Scale (mm) Sample Result Range.

## Data Availability

The data that support the findings of this study are available from the corresponding author, upon reasonable request.

## Abbreviations

The following abbreviations are used in this manuscript:

AAS	Atomic Absorption Spectrometry
AMR	Antimicrobial Resistance
AMX	Amoxicillin
BHI	Brain Heart Infusion
CAMHB-LHB	Cation-Adjusted Mueller–Hinton Broth Supplemented with Lysed Horse Blood
CCUG	Culture Collection University Of Gothenburg
CEQ	Catechin Equivalents
CFU	Colony Forming Unit
CHX	Chlorhexidine
CLSI	Clinical and Laboratory Standard Institute
DL	Detection Limit
DPPH	2,2-Diphenyl-1-Picrylhydrazyl
FC	Folin–Ciocalteu’s
GAE	Gallic Acid Equivalents
HACEK	Haemophilus *Actinobacillus actinomycetemcomitans* (*Aggregatibacter actinomycetemcomitans*), *Cardiobacterium hominis*, *Capnocytophaga* spp. *Eikenella corrodens* and *Kingella kingae*.
HRP	Horseradish Peroxidase
HTM	Haemophilus Test Medium
IZ	Inhibition Zone
JC	Jasmine Cooperative
K	Potassium
LtxA	leukotoxin
MBC	Minimum Bactericidal Concentration
Mg	Magnesium
MGHF	Medical-Grade Honey-based Formulation
MH	Mueller–Hinton
MIC	Minimum Inhibitory Concentration
ND	Not Determined
RJ	Royal Jelly
RP-HPLC	Reversed-Phase High-Performance Liquid Chromatography
RSA	Radical-Scavenging Activity
RSD	Relative Standard Deviation
RT	Room Temperature
TFC	Total Flavonoid Content
TPC	Total Phenolic Content
TTC	Triphenyl Tetrazolium Chloride
Zn	zinc
